# Supplementary material for: Implantable batteryless device for on-demand and pulsatile insulin administration
Source: Nat Commun. 2017 Apr 13;8:15032. doi: 10.1038/ncomms15032 (PMC5399301; doi:10.1038/ncomms15032)
Supplement: Supplementary Information — Supplementary Figures, Supplementary Tables, Supplementary Methods, and Supplementary References [file ncomms15032-s1.pdf]

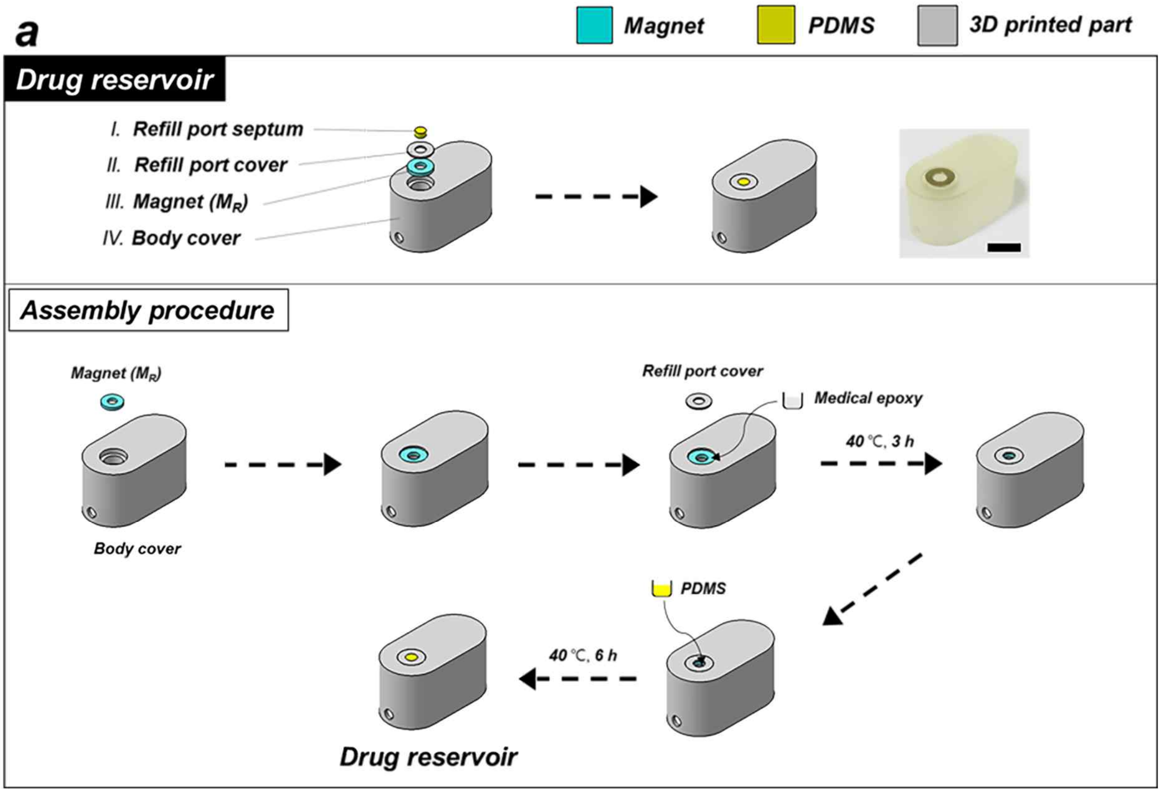
a
Magnet
PDMS
3D printed part
Drug reservoir
I. Refill port septum
II. Refill port cover
III. Magnet (M_R)
IV. Body cover
Assembly procedure
Magnet (M_R)
Body cover
Refill port cover
Medical epoxy
40 ℃, 3 h
PDMS
40 ℃, 6 h
Drug reservoir

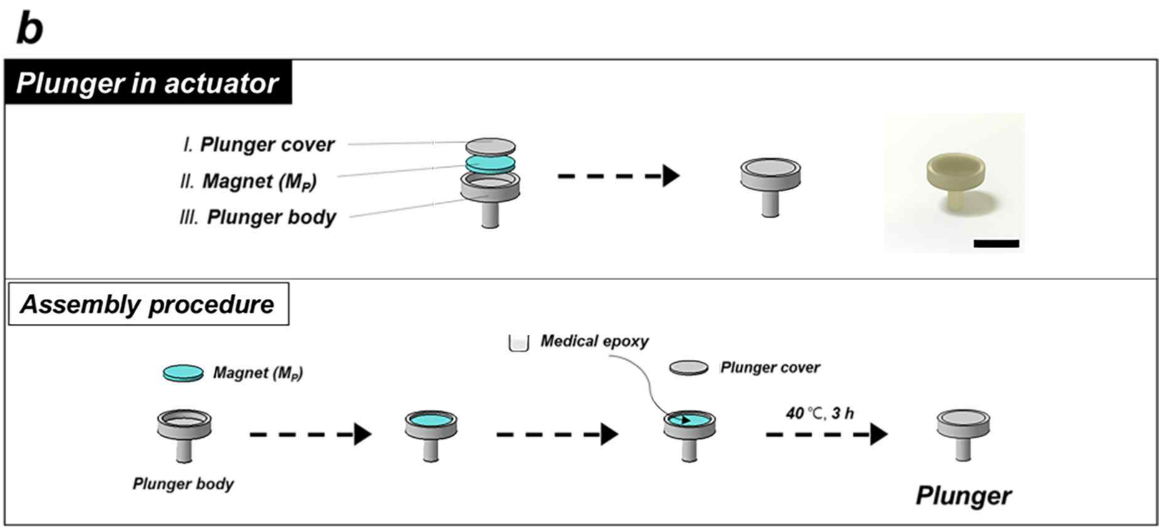
b
Plunger in actuator
I. Plunger cover
II. Magnet (M_P)
III. Plunger body
Assembly procedure
Magnet (M_P)
Plunger body
Medical epoxy
Plunger cover
40 ℃, 3 h
Plunger

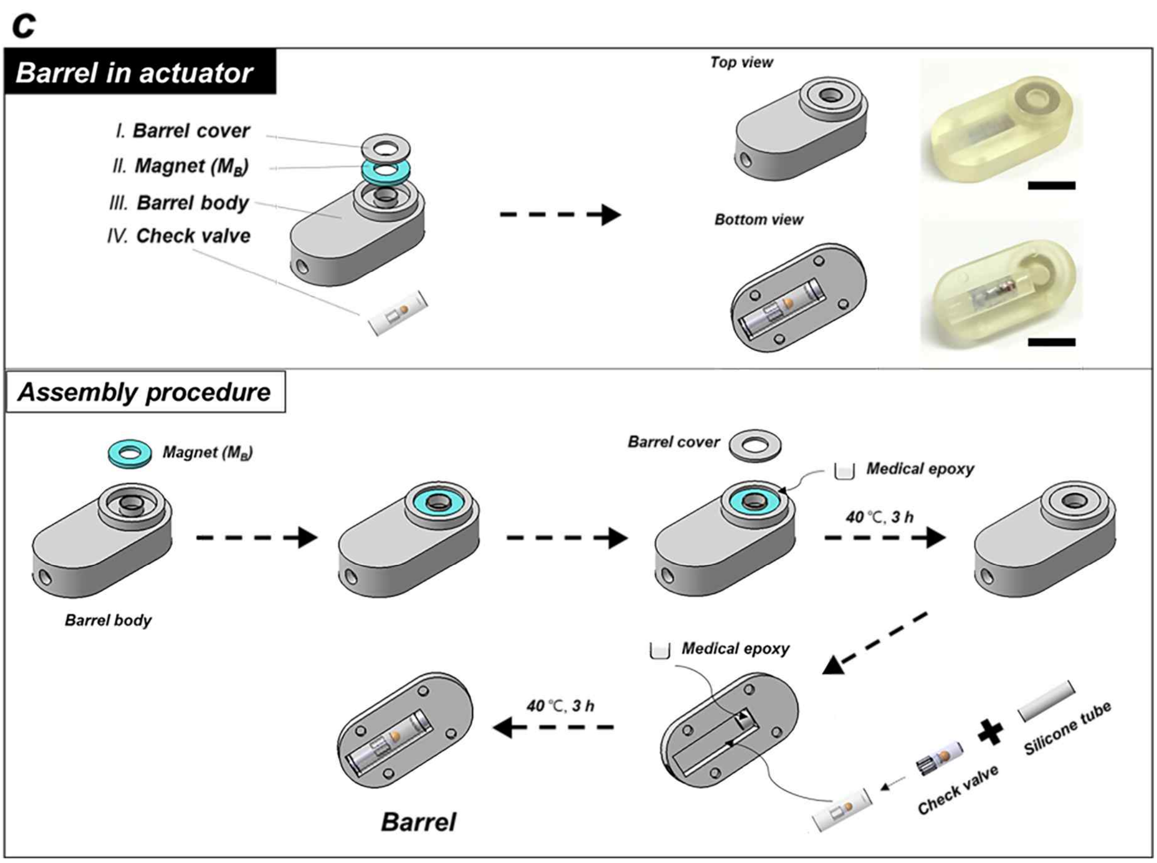
c
Barrel in actuator
I. Barrel cover
II. Magnet (M_B)
III. Barrel body
IV. Check valve
Top view
Bottom view
Assembly procedure
Magnet (M_B)
Barrel body
Barrel cover
Medical epoxy
40 ℃, 3 h
Medical epoxy
40 ℃, 3 h
Silicone tube
Check valve
Barrel

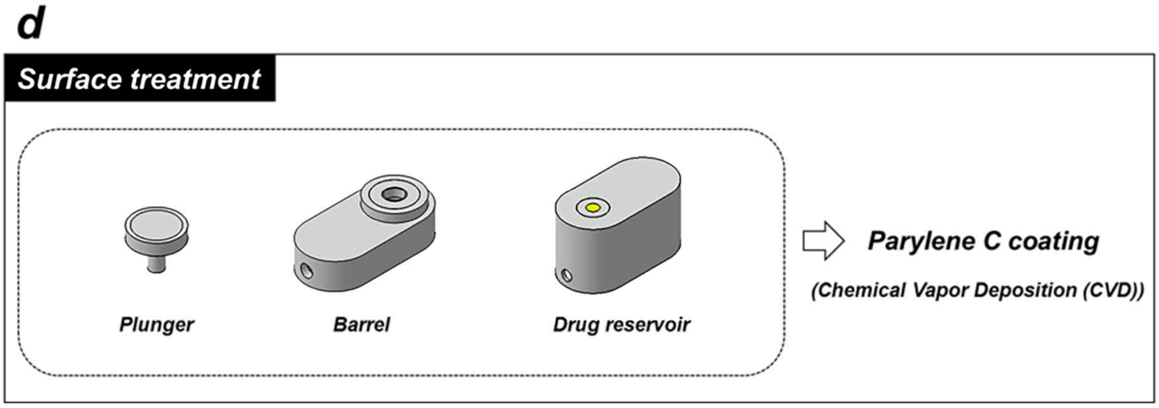
d
Surface treatment
Plunger
Barrel
Drug reservoir
Parylene C coating
(Chemical Vapor Deposition (CVD))

3

e

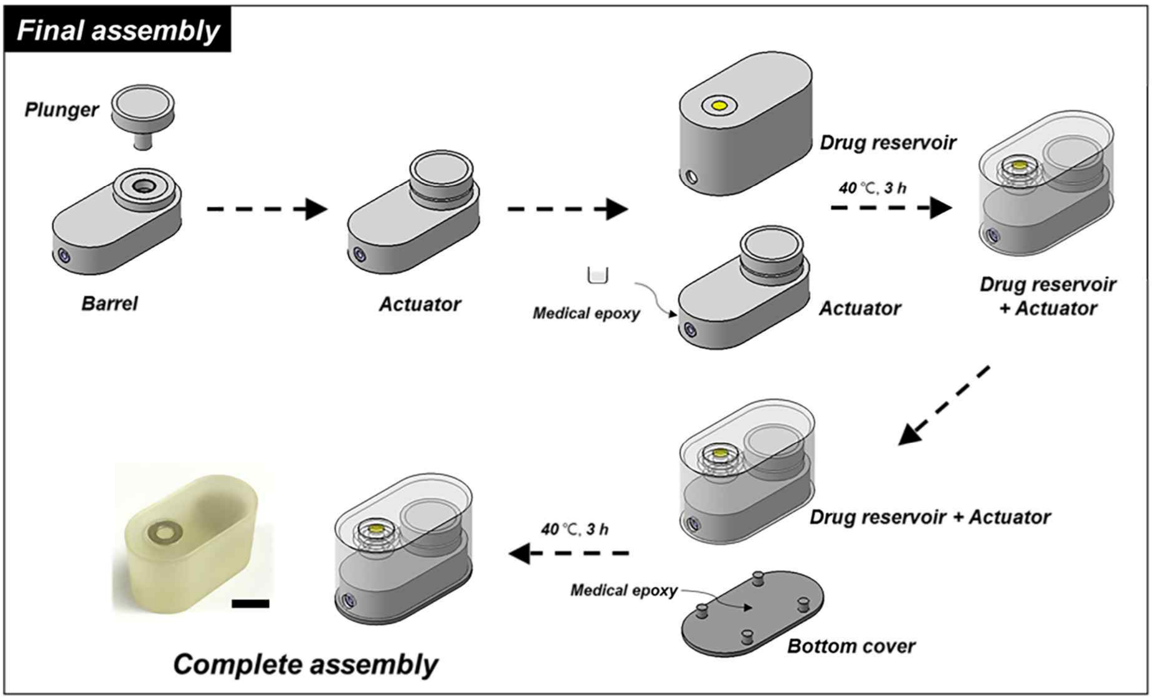
Final assembly
Plunger
Barrel
Actuator
Drug reservoir
40 ℃, 3 h
Medical epoxy
Actuator
Drug reservoir
+ Actuator
Drug reservoir + Actuator
40 ℃, 3 h
Medical epoxy
Bottom cover
Complete assembly

f

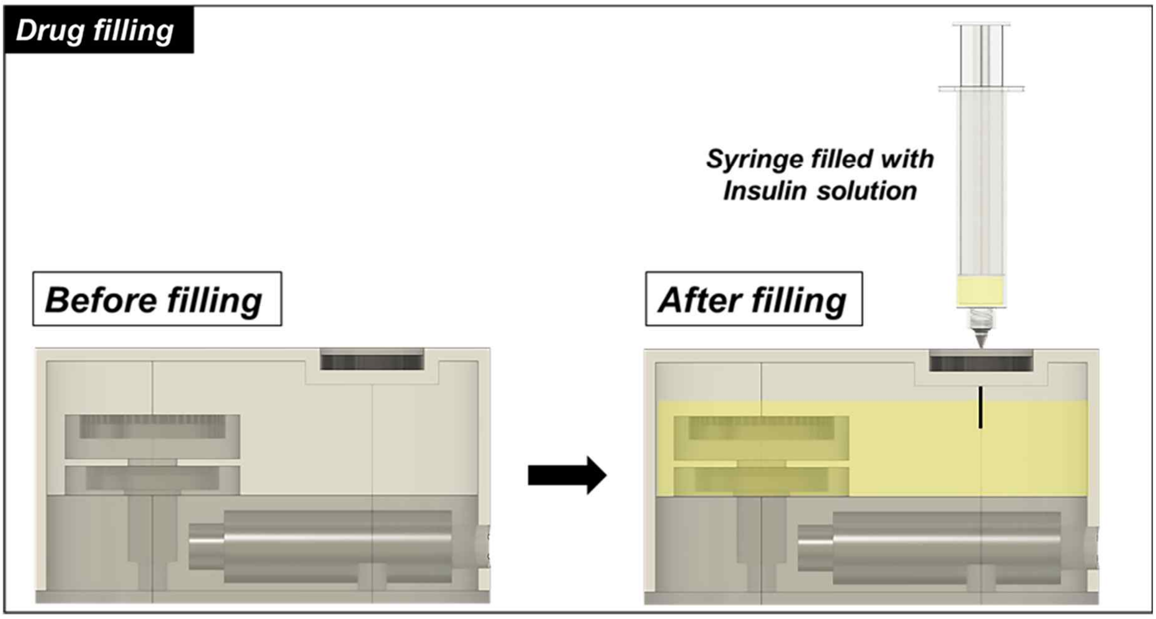
Drug filling
Syringe filled with
Insulin solution
Before filling
After filling

3

Supplementary Figure 1 Detailed description of the MDP fabrication procedures

The MDP consists of two distinct units: a drug reservoir and an actuator. (a) To prepare the drug reservoir, a body cover was assembled with a reservoir magnet of donut-shape (M_R), which was then assembled and bonded with a refill port cover. The hole formed in the refill port was then filled with Polydimethylsiloxane (PDMS) to prepare a septum. (b) To prepare the plunger in the actuator, a plunger body was assembled with a plunger magnet of coin-shape (M_P), which was then assembled and bonded with the plunger cover. (c) To prepare the barrel in the actuator, a barrel body was assembled with a barrel magnet of donut-shape (M_B), which was then assembled and bonded with a barrel cover. The resulting product was then assembled and bonded with a check valve inserted into a silicone tube. (d) The plunger, barrel and drug reservoir were coated with Parylene C by chemical vapor deposition. (e) The plunger was assembled with the barrel to form the actuator unit. Then, the actuator was assembled and bonded with the drug reservoir, which was then bonded with a bottom cover to complete the assembly. (f) The MDP was filled with 1.2 ml of an aqueous solution of insulin with a 30G syringe needle through the septum port into the drug reservoir. The schematic was drawn with Solidworks (Dassault Systemes, USA).

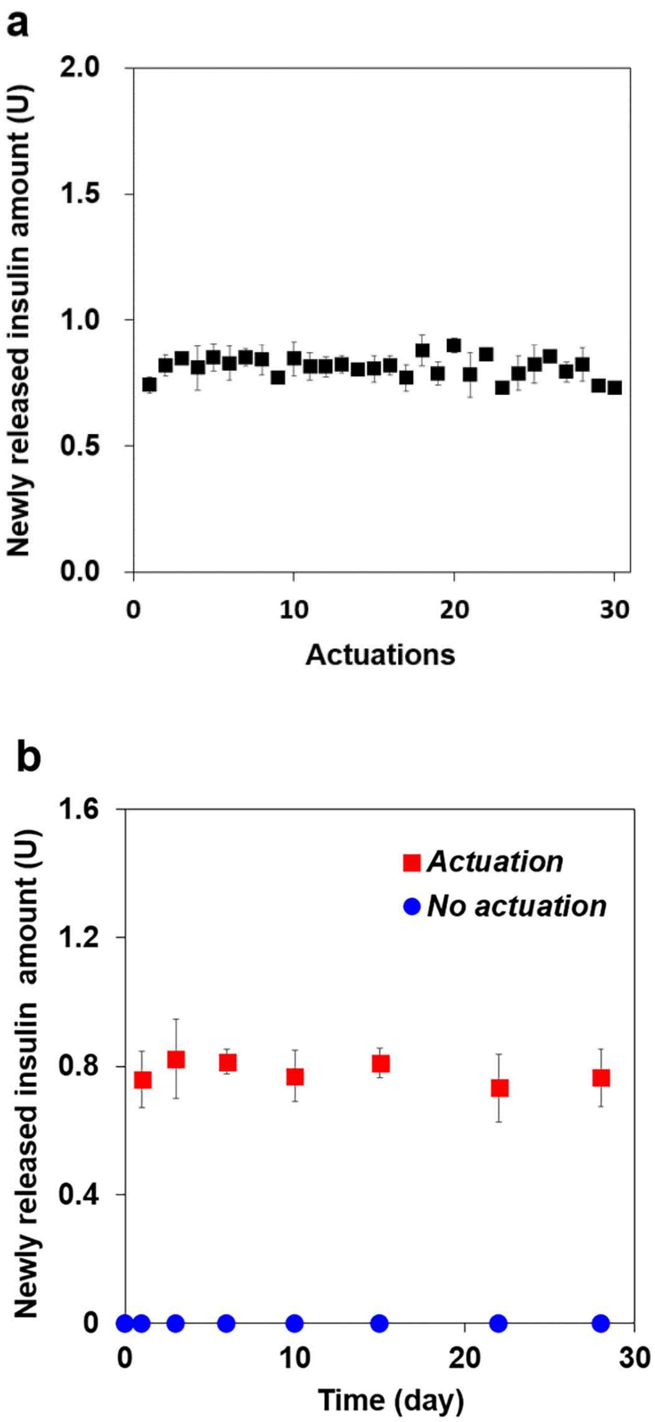
a
Newly released insulin amount (U)
2.0
1.5
1.0
0.5
0.0
0
10
20
30
Actuations
b
Newly released insulin amount (U)
1.6
1.2
0.8
0.4
0
0
10
20
30
Time (day)
Actuation
No actuation

3

Supplementary Figure 2 Insulin release profiles of the MDP

(a) Figure 2(a) and (b) Figure 2(b) were replotted to show the amount of newly released insulin per actuation. The released amounts of insulin were highly reproducible and were (a) 0.81 ± 0.04 U and (b) 0.80 ± 0.09 U per actuation. When there was no actuation, insulin was not detected with the measurement method employed in this work and thus, statistical analysis was not performed. Error bars are s.d.

a

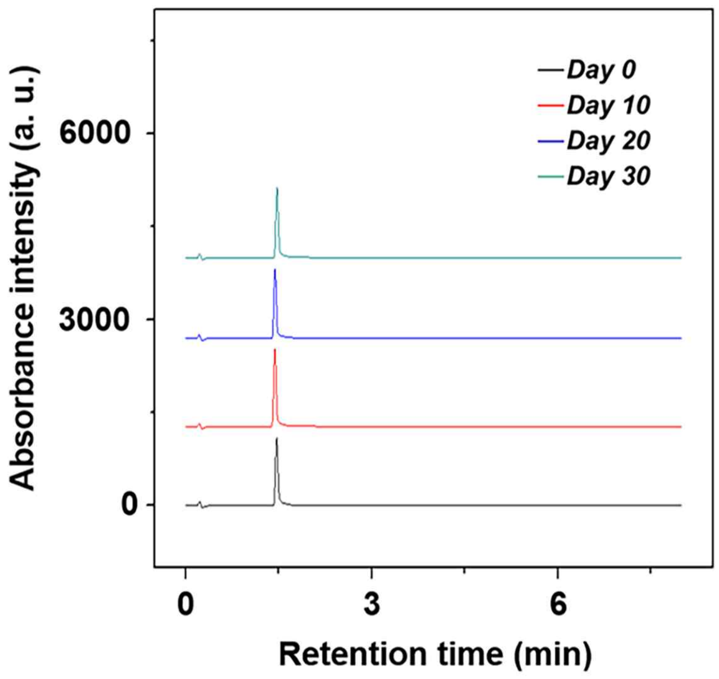
Day 0
Day 10
Day 20
Day 30
Absorbance intensity (a. u.)
6000
3000
0
0
3
6
Retention time (min)

b

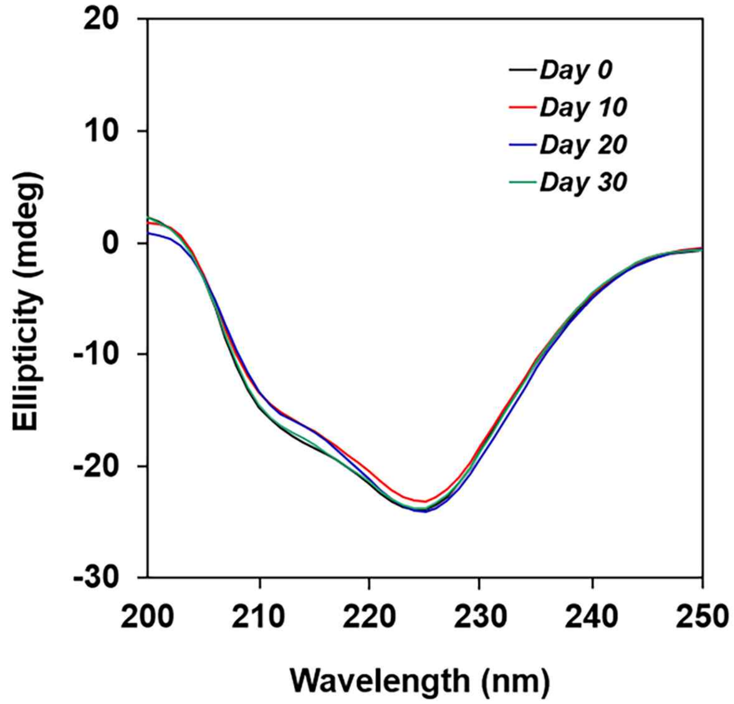
Day 0
Day 10
Day 20
Day 30
Ellipticity (mdeg)
20
10
0
-10
-20
-30
200
210
220
230
240
250
Wavelength (nm)

c

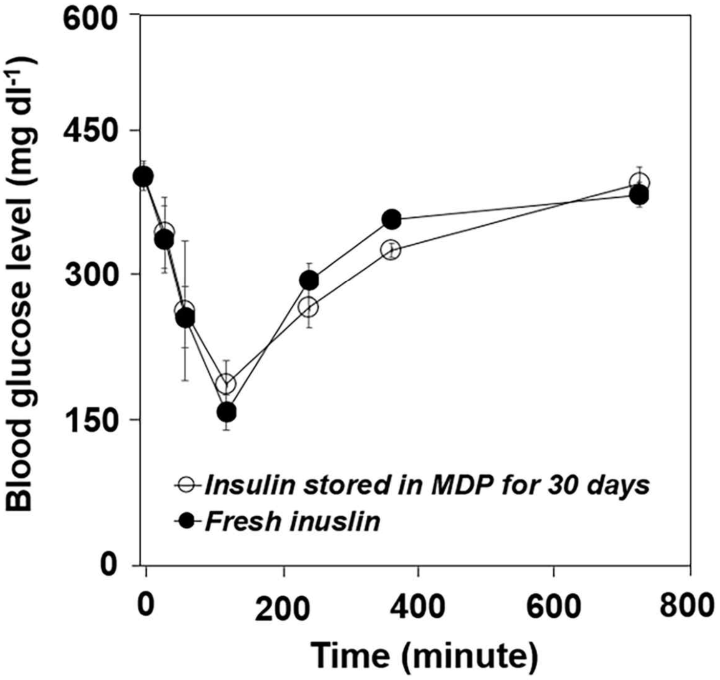

600
450
300
150
0
Blood glucose level (mg dl-1)
0
200
400
600
800
Time (minute)
Insulin stored in MDP for 30 days
Fresh inuslin

Supplementary Figure 3 Stability evaluation of insulin

An insulin solution (109 U/ml) in PBS (pH 7.4) was stored in the MDP at 37 °C for periods of 0, 10, 20 and 30 days and analyzed by RP-HPLC and circular dichroism (CD) spectroscopy. (a) The RP-HPLC analysis was performed following the same procedure described in Figure 2 in the main text. The uniform intensity and constant retention time of insulin among the tested incubation periods indicate that the insulin stored in the MDP was retained without aggregation or degradation until day 30 of incubation at body temperature. (b) CD spectra were obtained using a CD spectrometer (J-810, Jasco, Japan). A quartz cuvette with a 1-mm path length was used, and spectra were scanned at 200–250 nm. The CD spectra were seen to be similar among the tested incubation periods to a large extent, implying that for most of insulin in the MDP, the secondary structure remained unchanged after 30 days of incubation at body temperature. (c) A fresh insulin solution and a solution extracted from the MDP after incubation for 30 days were subcutaneously injected to diabetic rats, respectively ($n = 4$; fresh insulin, $n = 4$; insulin stored in the MDP for 30 days). A fresh insulin solution was prepared at the same concentration (109 U/ml) as the one initially used for stability test with the MDP and the same volume (7.4 µl) of each of the solutions was injected. Between two groups, the profiles of blood glucose level were similar without statistically significant difference ($P > 0.05$), implying that most of insulin stored in the MDP for 30 days could still effectively lower the blood glucose level. Error bars are s.d.

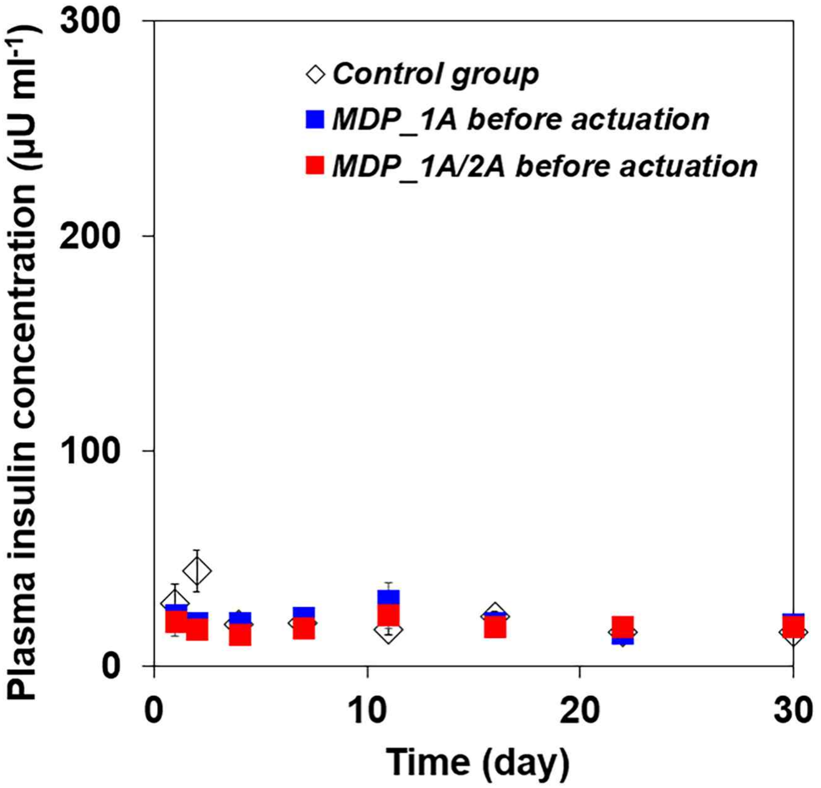

Supplementary Figure 4 Leak test results of the MDP

The plasma insulin concentration was measured immediately before actuation and compared to that of the control group (i.e., diabetic rats without treatment) (n = 4 ; control, n = 4 ; MDP_1A, n = 4 ; MDP_1A/2A). Error bars are s.d.

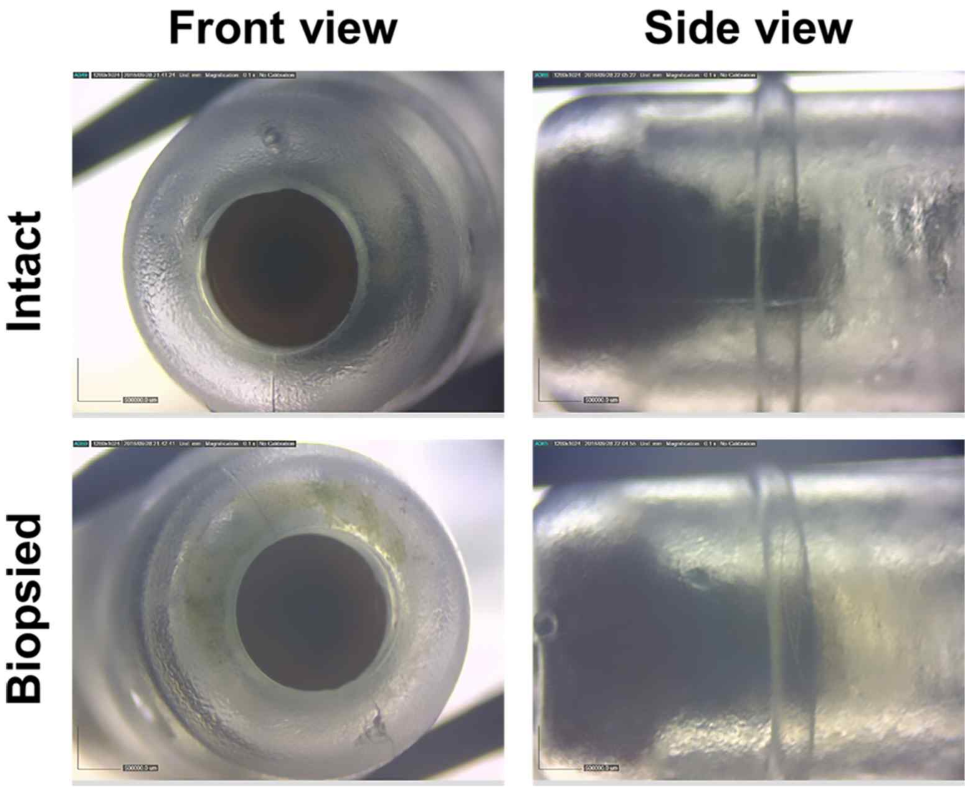

Supplementary Figure 5 Representative images of the intact and extracted valves

The extracted valve was obtained from the MDP biopsied at 60 days after implantation. No sign of clogging was seen with the valve in the implanted MDP.

a

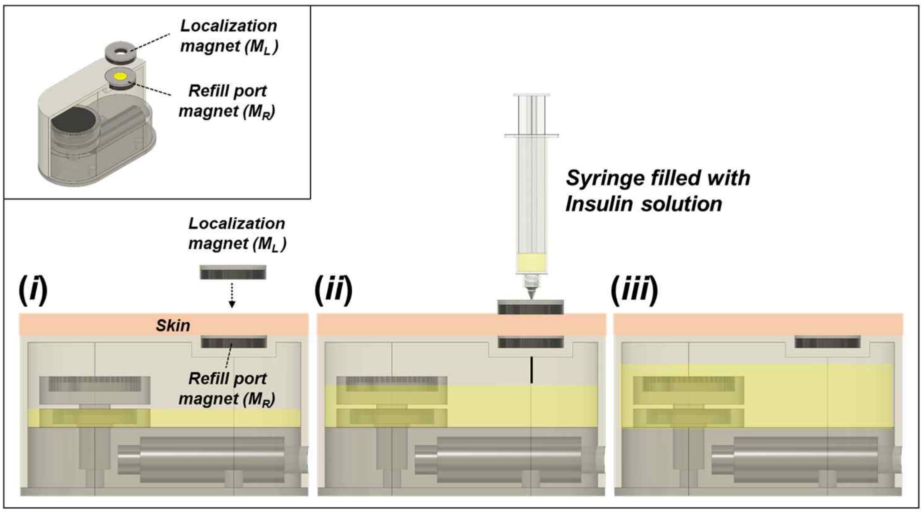
Localization magnet (M_L)
Refill port magnet (M_R)
Syringe filled with Insulin solution
Localization magnet (M_L)
(i)
(ii)
(iii)
Skin
Refill port magnet (M_R)

b

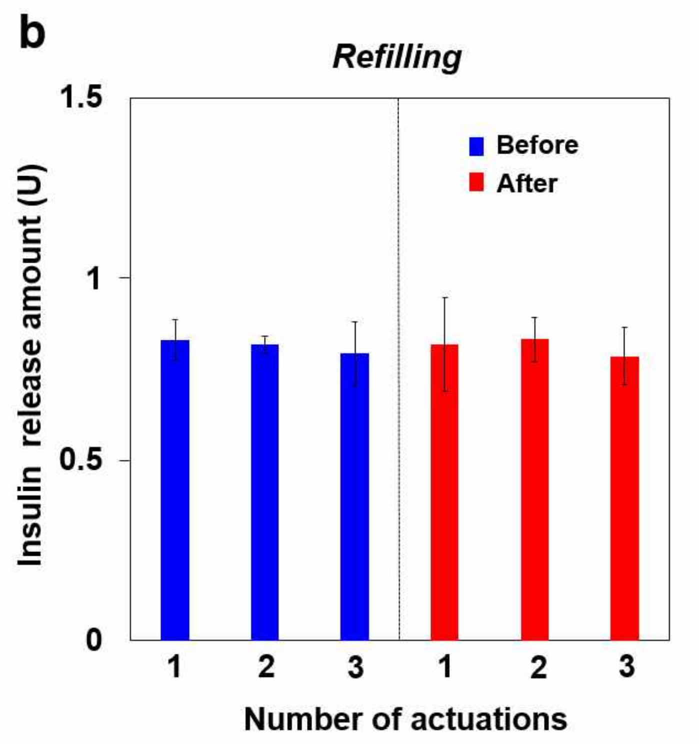
Refilling
Before
After
Insulin release amount (U)
0
0.5
1
1.5
1
2
3
1
2
3
Number of actuations

c

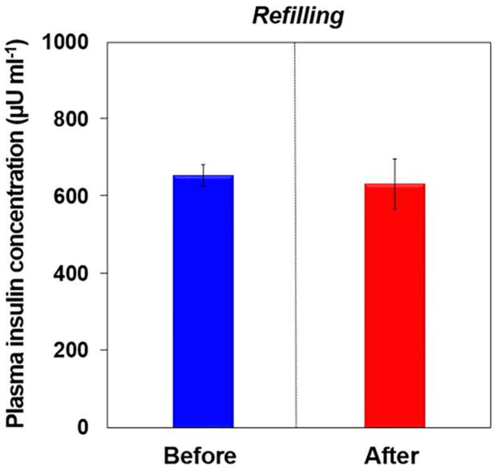
Refilling
Plasma insulin concentration (μU ml-1)
1000
800
600
400
200
0
Before
After

d

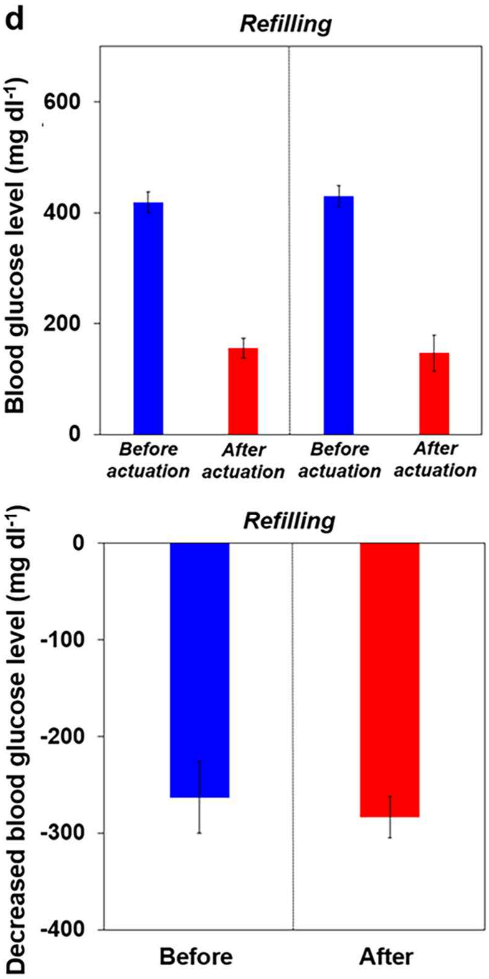
Refilling
Blood glucose level (mg dl-1)
600
400
200
0
Before actuation
After actuation
Before actuation
After actuation
Refilling
Decreased blood glucose level (mg dl-1)
0
-100
-200
-300
-400
Before
After

Supplementary Figure 6 Reproducibility assessment of the MDP after a refilling procedure

For this evaluation, we first simulated consumption of insulin by intentionally withdrawing 0.4 ml of the insulin solution in the drug reservoir and then injected the same volume of fresh insulin solution through the septum port of the MDP using a 30G needle. (a) Schematic description of the refilling procedure. *(i)* The refill port is located with a localization magnet (M_L) with a donut shape and polarity opposite that of M_R; *(ii)* a syringe needle is inserted through the refill port, and 0.4 ml of insulin solution (109 U/ml) is injected; and *(iii)* the drug reservoir is filled with the insulin solution. (b) *In vitro* insulin release profiles before and after a refilling procedure. The MDP was fully immersed in phosphate-buffered saline (PBS; pH 7.4) at 37 °C. Initially, three actuations were conducted at 10-min intervals. After each actuation, the amount of released insulin was measured. Then, a refilling procedure was performed, and the experiments were repeated. Three MDPs were tested for this experiment. Error bars are s.d. (c, d) *In vivo* profiles of plasma insulin concentration and glucose level in blood before and after the refilling procedure (n = 3). The refilling procedure was performed while the MDP was implanted in streptozotocin (STZ)-induced diabetic rats. The septum port was found from outside of skin using a localization magnet (M_L) with a polarity opposite that of M_R. To measure the plasma insulin concentration, blood was collected at 60 min after actuation. The blood glucose levels were measured at -1 and 120 min after actuation. Error bars are s.d.

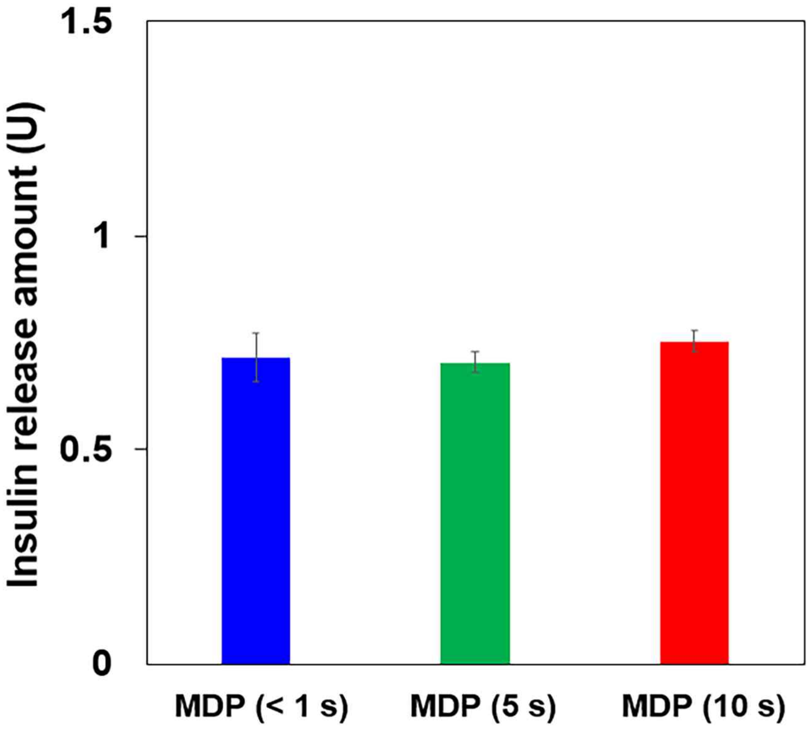

Supplementary Figure 7 Reproducibility assessment of the MDP with varying the periods for the external device (M_E) application

Under the *in vitro* drug release experimental condition depicted in Fig. 2, the external device was applied and removed to the MDP during the periods of < 1 s, 5 s and 10 s, respectively (i.e., MDP (< 1 s), MDP (5 s) and MDP (10 s), respectively). The results revealed that the delivered doses of insulin per actuation were quite similar regardless of the period for the external device application. Three distinct MDPs were tested for each period for the external device application. Error bars are s.d.

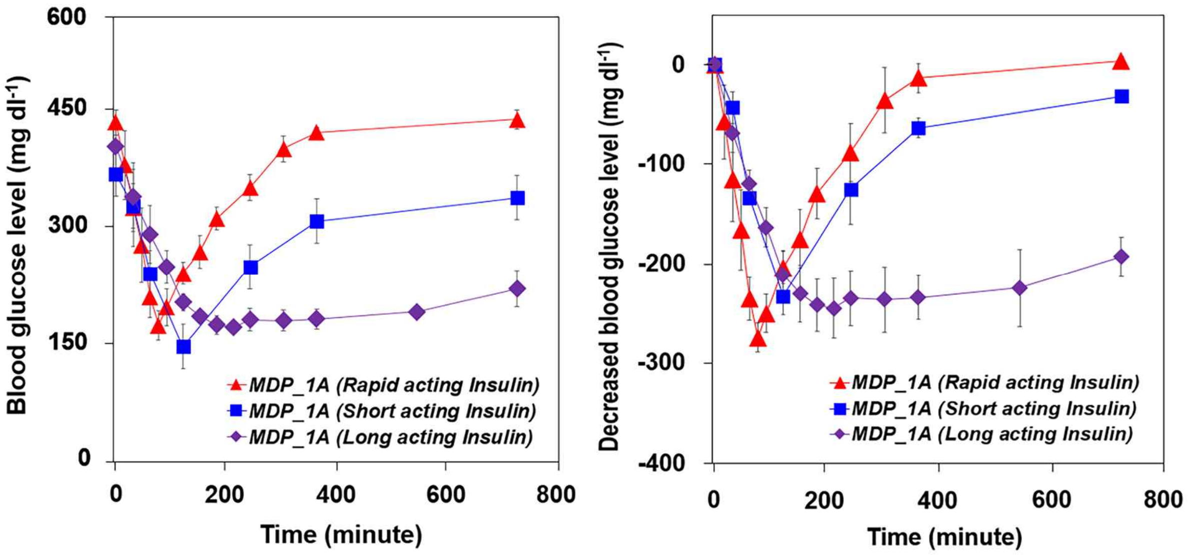

Supplementary Figure 8 Profiles of blood glucose level obtained with the three different insulin formulations

In addition to a short acting insulin mainly used in this study (i.e., MDP_1A (short acting insulin)), we additionally prepared the MDP filled with a rapid acting or long acting insulin formulation (NovoRapid[1] or Lantus[2], respectively) to give the animal groups of MDP_1A (rapid acting insulin) or MDP_1A (long acting insulin), respectively. At 1 day after the MDP was implanted in STZ-induced diabetic rats, the blood glucose level was obtained at -1 to 720 min after insulin administration by a single actuation of the MDP. A similar dose of insulin was administered for all experimental groups (rapid acting: 0.74 U; short acting: 0.80 U; and long acting 0.74 U) . For each animal group, 4 rats were employed for statistics. Error bars are s.d. For all formulations, the decrease in blood glucose level was apparent after actuation and as expected, a specific profile of blood glucose level was observed for each type of the insulin formulations filled in the MDP. For rapid acting insulin, the blood glucose level decreased and increased back more rapidly. For long acting insulin, the blood glucose level dropped relatively slowly and this lowered level was maintained for a longer period.

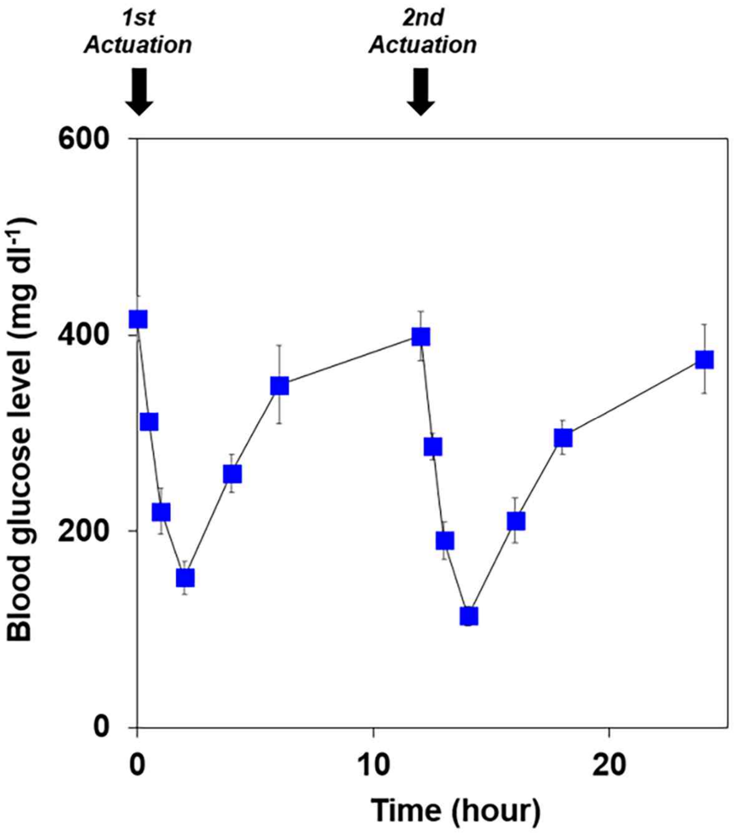

Supplementary Figure 9 Profiles of blood glucose level with multiple daily actuations of the MDP

To give an insight of insulin delivery after each of the meal times, the MDP was actuated twice with an interval of 12 h after implantation. After the first actuation, a glucose level dropped and increased back to a high level as expected with diabetic rats, which was observed to be repeated in a similar pattern after the second actuation (n=4). Error bars are s.d.

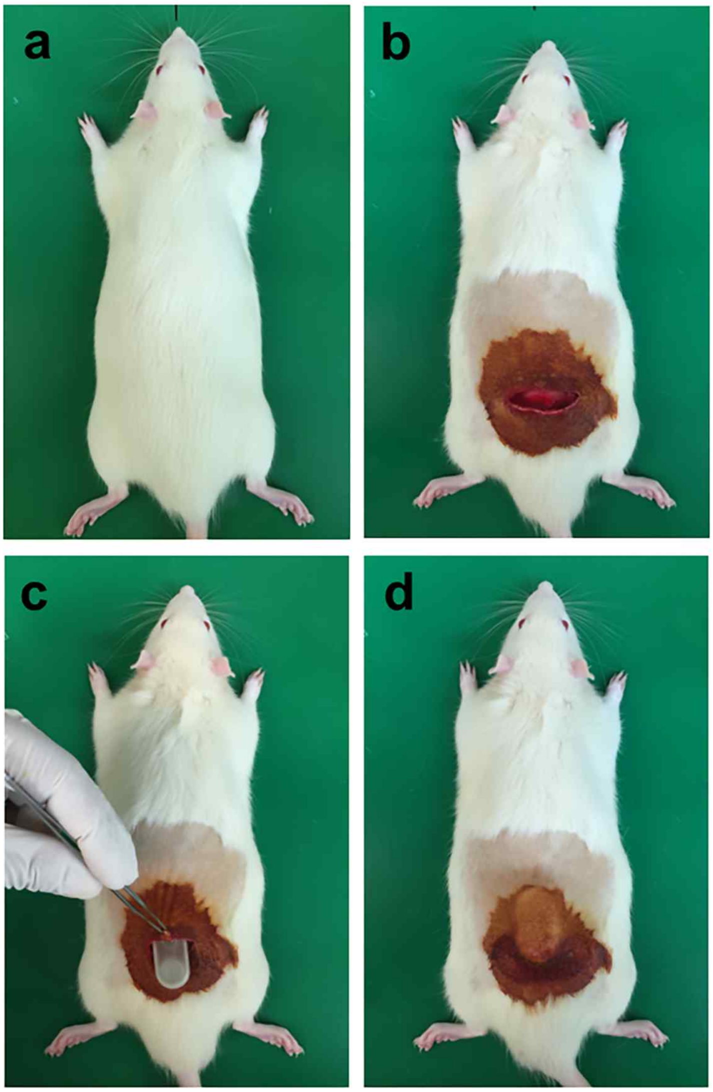

Supplementary Figure 10 Surgical procedure for MDP implantation

(a) A diabetic rat was anesthetized via intraperitoneal injection of a mixture (1 ml/kg) of Zoletil® 50 and Rompun® (1:1 v/v). (b) The dorsal area was shaved and sterilized with betadine. (c) A 4–5 cm skin incision was made, and the MDP was implanted into the dorsal subcutaneous pocket. (d) The wound was closed with a surgical suture and disinfected with betadine.

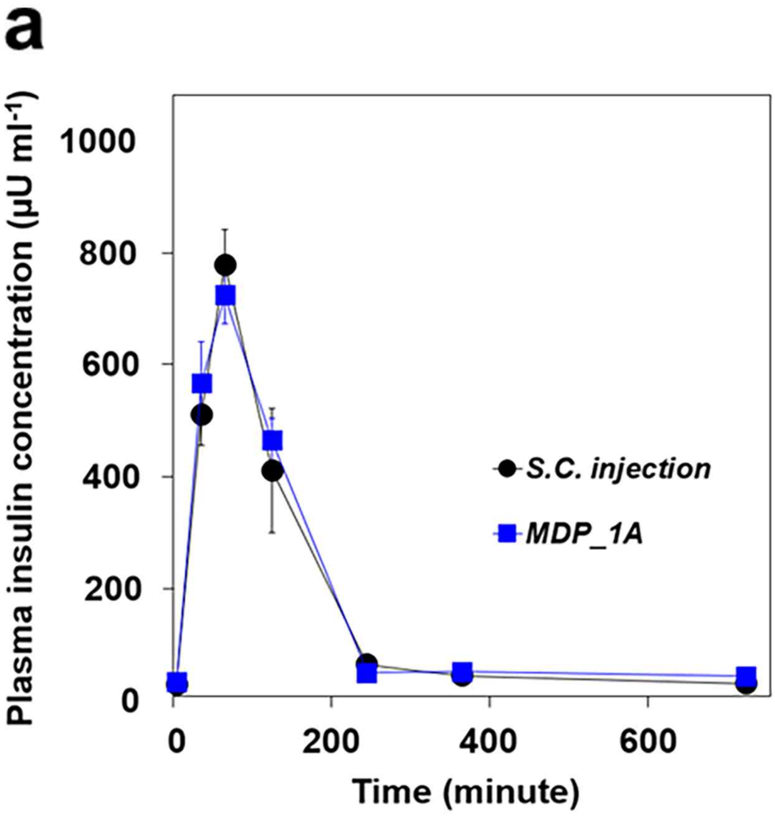
a
Plasma insulin concentration (μU ml-1)
1000
800
600
400
200
0
0
200
400
600
Time (minute)
S.C. injection
MDP_1A

b

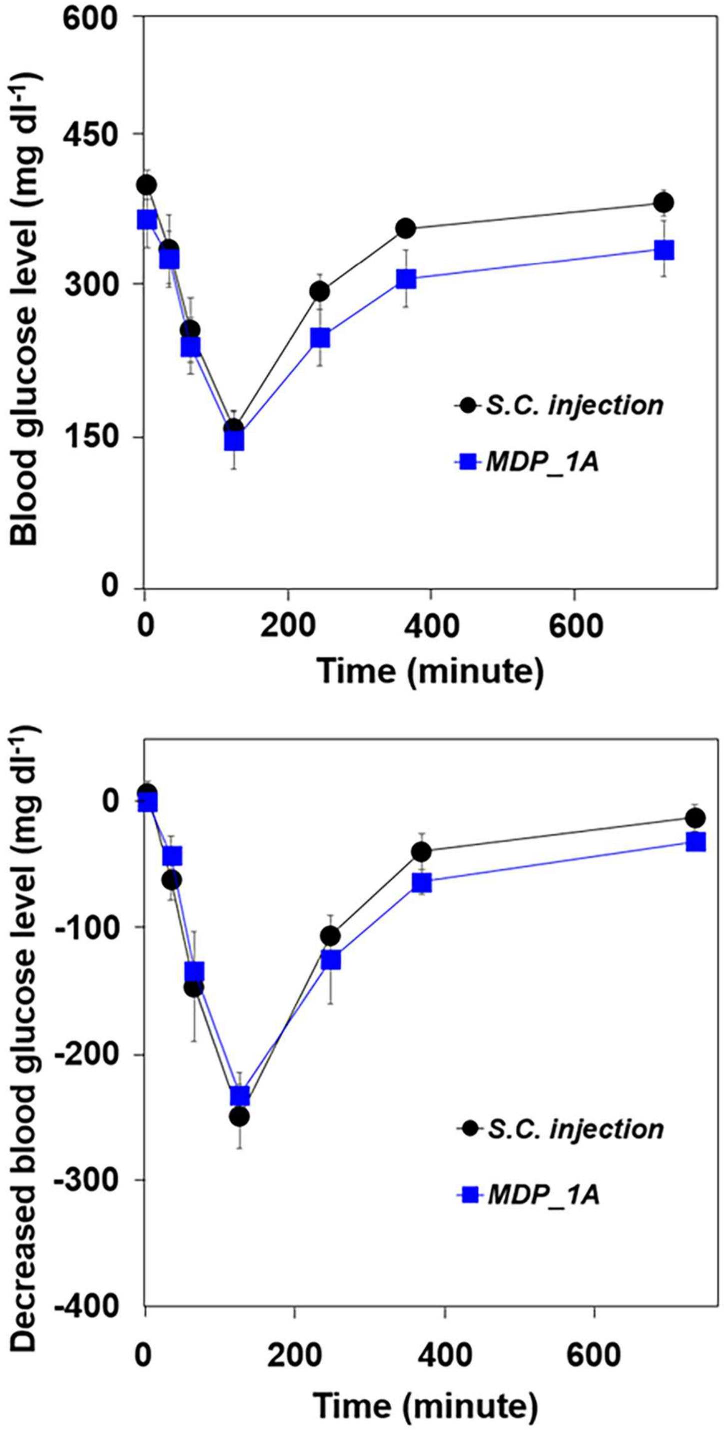
600
450
300
150
0
Blood glucose level (mg dl-1)
0
200
400
600
Time (minute)
S.C. injection
MDP_1A
0
-100
-200
-300
-400
Decreased blood glucose level (mg dl-1)
0
200
400
600
Time (minute)
S.C. injection
MDP_1A

Supplementary Figure 11 Short-time profiles of plasma insulin concentration and blood glucose level

(a) The plasma insulin concentration was measured by sampling blood at -1, 30, 60, 120, 240, 360 and 720 min after insulin administration. The maximum insulin concentration occurred at 60 min in both the S.C. injection and MDP groups. Error bars are s.d. There was no statistically significant difference between two groups ($P > 0.05$). (b) The blood glucose level was measured at -1, 30, 60, 120, 240, 360 and 720 min after insulin administration. The maximum decrease in glucose level occurred at 120 min in both the S.C. injection and MDP groups ($n = 4$; S.C. injection, $n = 4$; MDP_1A). Error bars are s.d. There was no statistically significant difference between two groups ($P > 0.05$).

Supplementary Table 1 Actuation ability of the MDP according to the gap between the external device and MDP

The MDP could be actuated at gaps of up to 3 mm and in this range, the MDP could infuse the same amount of insulin, as observed in our *in vitro* performance test. To be actuated with the skin gap of 1 mm, the MDP herein needs an external magnet with 3000 G. Therefore, considering the range of a magnetic field available in a regular life style (< 2 G)[3], a chance for an accidental activation of the MDP is expected to be not high.

Gap between the external device and MDP (mm)	Actuation ability*
0	Y
1	Y
1.5	Y
2	Y
2.5	Y
3	Y
3.5	N

* Y : actuated; N : not actuated

Supplementary Table 2 Insulin amount left in fibrotic capsules

To assess the insulin amount possibly left in the fibrotic capsule, we biopsied the whole capsule including the MDP at two different days after implantation, i.e., at 16 days and 60 days, respectively (n = 5; 16 days, n = 4; 60 days). At each day, the MDP was actuated twice consecutively (1.6 U insulin delivery) and after 360 min, the biopsy was performed. From each of the biopsied capsule, we extracted the MDP and the surrounding capsule tissue was fully immersed in 5 ml of pH 7.4 PBS at 37 °C for 6 h. Then, the supernatant was analyzed with HPLC, as described in the Methods, to measure the insulin amount. Our results revealed that more than 97.5% of the total amount of delivered insulin was diffused out from the fibrotic capsule during the first 360 min after actuations.

Day	Insulin amount in fibrotic capsule (U)
16	0.032 ± 0.011
60	0.030 ± 0.008

Values ± s.d.

Supplementary Table 3 Inflammatory markers in plasma

Inflammatory markers in plasma, such as IL-1b, IL-6 and TNF-α, were measured at 30 and 60 days after MDP implantation (n = 3; 30 days, n = 3; 60 days). For all animals, elevation of inflammatory markers was not observed and their levels in plasma were not different from the ones with the control animal group (i.e., the animals without MDP implantation).

	IL-1b (pg ml^{-1})	IL-6 (pg ml^{-1})	TNF-α (pg ml^{-1})
Day 30	68 ± 10	53 ± 4.0	82 ± 11
Day 60	68 ± 4.0	56 ± 3.0	87 ± 8.2
Control	71 ± 8.6	60 ± 3.2	94 ± 3.0

Values ± s.d.

Supplementary Table 4 Amount of released insulin with varied catheter lengths

The MDP without a catheter (i.e., a catheter, 0 cm in length) and the ones connected with a catheter, 10 and 15 cm in length, respectively, were each actuated under the *in vitro* experimental condition, as depicted in Fig. 2. Although the amount of released insulin decreased with the catheter length, insulin could still be released in a reproducible manner with a catheter length of up to 15 cm and this was reported to be similar to an anatomical distance between the subcutaneous and intraperitoneal space in humans[4]. The result indicated a reproducible volume of liquid infused per actuation and thus, the dose of insulin could be accommodated by employing a proper concentration of insulin formulation to be filled in the drug reservoir.

Catheter length (cm)	Insulin release amount (U)
0	0.81 ± 0.04
10	0.74 ± 0.04
15	0.68 ± 0.04

Theoretical assessment of magnetic forces between the plunger and barrel magnets

The attraction force between the plunger and barrel magnets (M_P and M_B, respectively) was measured to be 1.25 N (Advanced force measurement 9830, Interface, USA), which could fix their position to not release insulin during the period of no actuation. With the mass of the plunger used in this study (9.8 x 10^{-4} kg), therefore, the acceleration needed to overcome this attraction force and move the plunger was calculated to be 1.28 x 10^{3} m s^{-2}, which was more than 100 times larger than gravitational acceleration (9.8 m s^{-2}). In this sense, a chance for an accidental movement of the plunger in the MDP herein is expected to be very low.

Supplementary References

1. Reynolds, N.A. & Wagstaff, A.J. Insulin Aspart. *Drugs* **64**, 1957-1974 (2004).
2. Wang, F., Carabino, J.M. & Vergara, C.M. Insulin glargine: a systematic review of a long-acting insulin analogue. *Clinical therapeutics* **25**, 1541-1577 (2003).
3. Hamdan, H. Measurements of ELF Electromagnetic Fields in Jordan Exposure Limits and Recommendations. *Dirasat: Engineering Sciences* **39**, (2014).
4. Thompson, J.S. & Duckworth, W.C. Insulin pumps and glucose regulation. *World J. Surg.* **25**, 523-526 (2001).
